# Identification of individual cells from z-stacks of bright-field microscopy images

**DOI:** 10.1038/s41598-018-29647-5

**Published:** 2018-07-30

**Authors:** Jean-Baptiste Lugagne, Srajan Jain, Pierre Ivanovitch, Zacchary Ben Meriem, Clément Vulin, Chiara Fracassi, Gregory Batt, Pascal Hersen

**Affiliations:** 1 Laboratoire Matière et Systèmes Complexes, UMR 7057 CNRS & Université Paris Diderot, 10 rue Alice Domon et Léonie Duquet, 75013 Paris, France; 2 Inria Saclay – Ile-de-France and Université Paris Saclay, 1 rue Honoré d’Estienne d’Orves, Bâtiment Alan Turing, Campus de l’Ecole Polytechnique, 91120 Palaiseau, France; 3 Laboratoire Biologie et Dynamique des Chromosomes, CNRS UMR 7212, Hôpital Saint-Louis, Paris, France; 4 ETH, Swiss Federal Institute of Technology, Zurich, Switzerland; 5 Institut Pasteur and CNRS, C3BI - USR 3756, 25–28 Rue du Docteur Roux, 75015 Paris, France

## Abstract

Obtaining single cell data from time-lapse microscopy images is critical for quantitative biology, but bottlenecks in cell identification and segmentation must be overcome. We propose a novel, versatile method that uses machine learning classifiers to identify cell morphologies from *z*-stack bright-field microscopy images. We show that axial information is enough to successfully classify the pixels of an image, without the need to consider in focus morphological features. This fast, robust method can be used to identify different cell morphologies, including the features of *E. coli*, *S. cerevisiae* and epithelial cells, even in mixed cultures. Our method demonstrates the potential of acquiring and processing Z-stacks for single-layer, single-cell imaging and segmentation.

## Introduction

Thanks to the development of microfluidics and microscopy, it is now possible to measure the dynamics of single cells over time^1,2^. In recent years, longitudinal time-lapse studies have emerged as key methods in quantitative biology and are essential to understand the dynamics of cellular processes^2–4^. However, a robust and efficient cell segmentation method is required to obtain high quality single cell traces^1,2^. Despite years of development, a universal method to segment cells from microscopy images has not yet been established. The numerous existing methods were designed for specific cell types and usually rely on specific morphological features (*e.g*., size, shape, fluorescent labeling). Although efficient for specific problems, these methods are not versatile, and usually fail when applied to different cell types or other experimental conditions. As a result, research groups design and tweak image analysis software^5–13^ to match their specific segmentation problem. This is a considerable waste of time and energy, and highlights the need for a simple, versatile strategy to segment cells, irrespective of experimental design or cellular characteristics.

Notably, segmentation critically depends on obtaining high-quality images with a constant focus that outlines the borders and main morphological features of the cell. This is an important constraint, which – in practice – requires periodic auto-focusing or a control system to automatically maintain perfect focus. Here, we propose a different segmentation strategy inspired by hyperspectral imaging. Instead of relying on the in-focus image, we systematically acquired multiple stacks of images around the focal plane (*i.e*. a *z*-stack) of various cells, and use the information contained within the full *z*-stack to identify the focal region of the cells in the images. The central idea is that cell contours, the cellular interior or any objects within the field of view do not have the exact same intensity profile throughout the *z*-dimension. Here, we show it is possible to train an algorithm using machine learning to classify *z*-pixels (the vector of light intensity along the *z*-axis for a specific pixel in the image) based on their focal signature. The method is simple, robust, can be run in real-time and – importantly – gives excellent results for *E. coli* (rod-shaped), *S. cerevisiae* (round), mammalian epithelial (HeLa) cells, and even a mixture of bacteria and yeast cells.

## Results

**Figure 1:**
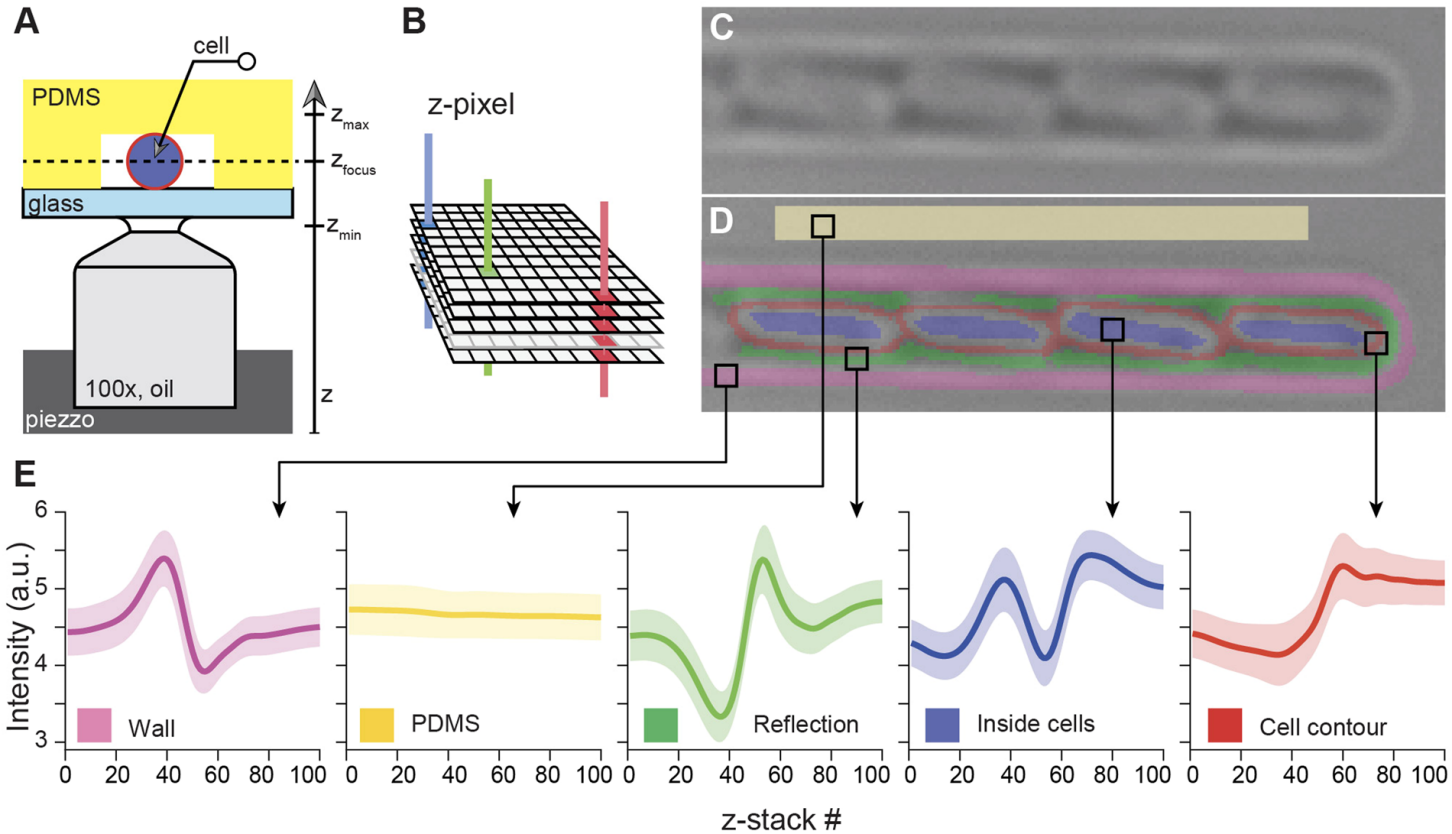
Principle of *z*-stack segmentation. (**A**) A piezo-driven system is used to quickly and precisely move the objective and acquire a stack of images below, at and above the focal plane (*z*-stack). (**B**) Acquisition of bright field *z*-stacks provides the focal signature of every *z*-pixel. (**C**) *E. coli* cells (~1 µm long, observed with a 100x objective) are cultured in a microfluidic device designed to keep them in lines. (**D**) The graphical user interface is used to define different classes of object by directly drawing them. Each *z*-pixel in the image contains a profile of intensity as a function of the *z*-stack position. (**E**) Average *z*-pixel profiles obtained from the example shown in (**C**,**D**), demonstrating that different classes have different focal signatures. The shaded areas are +/− one standard deviation. From this training dataset and the definition of classes, it is possible to classify each pixel in a *z-*stack into one of the classes.

We used a Piezzo drive (PIFOC, PI) to acquire *z*-stacks containing 100 images, 100 nm apart, of *E. coli* using a 100x oil objective (UPlanFL 1.3NA) and CoolSNAP HQ2 camera with a resolution of 1040 × 1392 pixels (Fig. 1A,B). *E. coli* cells were loaded into a microfluidic device where they were cultured in narrow chambers (Fig. 1C). A graphical user interface (GUI, Supplementary Text) was developed to manually label a training dataset by defining regions of interest (classes), such as the interior of the cell and its contours, then a machine learning classifier was trained on this dataset (see Supplementary Text). Importantly, the algorithm was not trained using the morphological features in the (*x–y*) plane – as it is classically done – but only a representative set of *z*-pixels (Fig. 1B) for each class of objects that the user wants to identify and segment in the image. Indeed, many such *z*-pixels can be found in a *z*-stack of a monolayer since it usually contains tens to a few hundred *E. coli* and any 10 × 10 pixels area (representing the typical surface of a cell) contains as many as 100 different profiles. Principal component analysis was used to reduce the dimensionality of the problem from 100 dimensions (if all *z*-positions in the stack are considered) to a lower dimension space (typically between 5 and 20 dimensions, see Supplementary Text), that enabled separation of the different user-defined classes (Fig. 1D,E). In practical terms, we used Matlab and its Support Vector Machine library (*fitcsvm* package), to perform training and class identification (see Supplementary Fig. S1). The classification of a *z*-pixel does not depend on the classification of other *z*-pixels, so the parallelization of the prediction process is straightforward.

**Figure 2:**
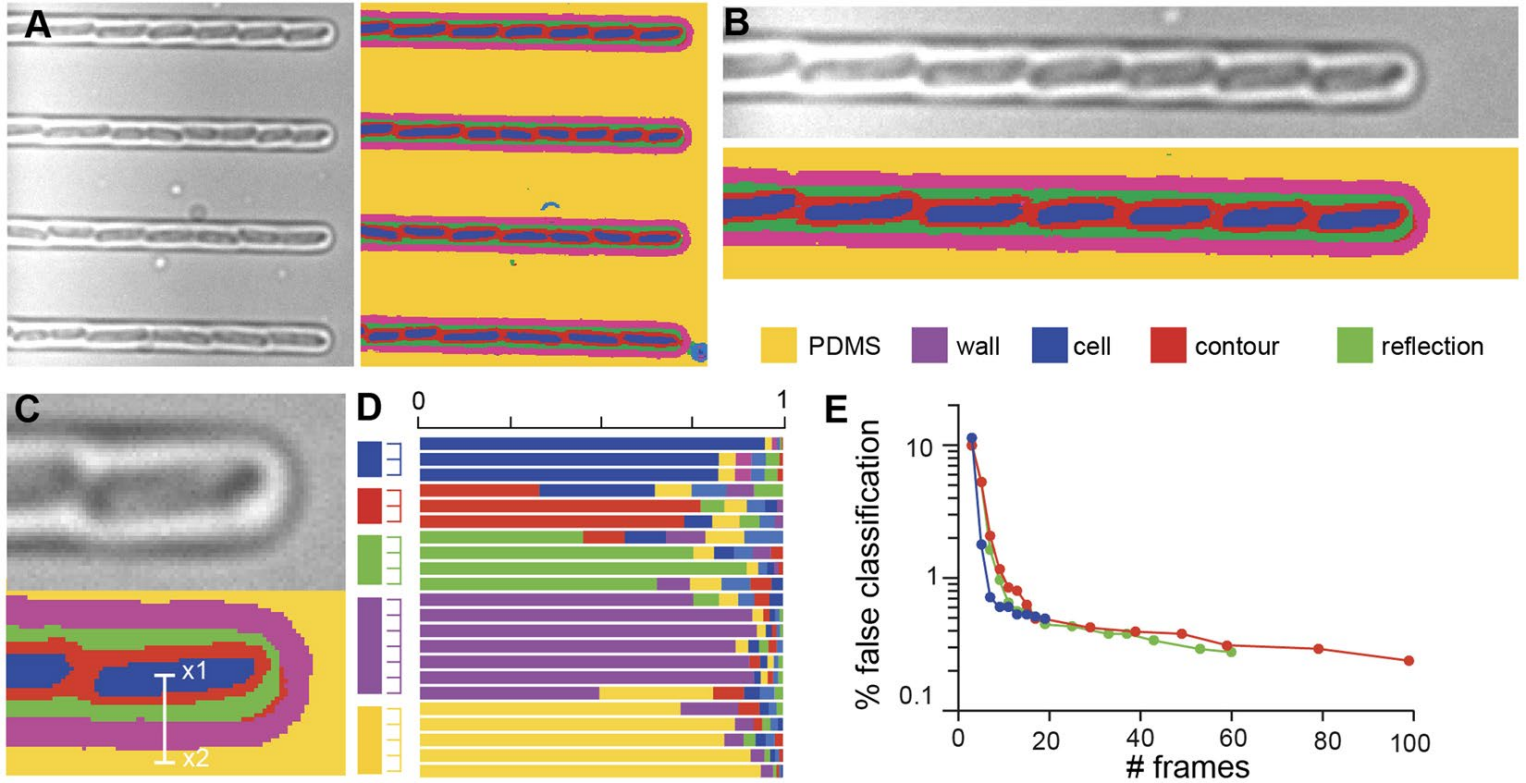
SVM classification enables robust, precise detection of cell features. (**A**) Applying the procedure described in Fig. 1, we can attribute *z*-pixels to different classes that are freely defined by the user (*e.g*. PDMS, microfluidic wall, cell interior, cell contour, halo between cells, microfluidic wall). (**B**,**C**) High-magnification image showing perfect classification of the interior and contours of *E. coli* cells. (**D**) Normalized confidence scores for each pixel of the *x1-x2* line shown in (**C**) The score of each class is computed as a *softmax* function (see Supplementary Information). (**E**) Validation of the method as a function of the number of frames used to identify the different object classes (red: evenly spaced images; blue: manually selected images; green: logarithmically spaced images). Irrespective of how the frames are chosen, misclassification was lower than 1% for a *z*-stack containing as few as 10 frames.

After training, we acquired another *z*-stack and used the SVM to classify the pixels. As shown in Fig. 2, the different parts of an image of *E. coli* cells were correctly identified with neither post-processing nor user intervention (see also Supplementary Fig. S2). The cells were detected, and it was even possible to locate and classify the cell contour, cell interior and microfluidic chamber with excellent fidelity. Therefore, this method is markedly more powerful than classic segmentation, since it enables identification of more than one type of structure, without any *a priori* knowledge of their morphological features in the focal plane. Moreover, Fig. 2C shows how automatic labeling of a pixel is associated with a confidence score that can be used to assess the quality of the classification and further refine cell segmentation (see Supplementary Text, Supplementary Figs S2–S6). Although it’s often interesting to define several classes to identify important objects on the image, classification of cells only requires two classes: “cell” and “not cell” (see supplementary text). The number of images can also be decreased while achieving good performance; the method gave excellent identification scores (classification error less than 1%) if the *z*-stack contained at least seven images (Fig. 2D, Supplementary Fig. S3). On our computer system (20-cores Xeon, DELL), training the SVM on a typical dataset took from 15 min to 1 hour at most, and a little longer than 1 minute to attribute the pixels to their classes in a 1392 × 1040 × 100 *z*-stack.

**Figure 3:**
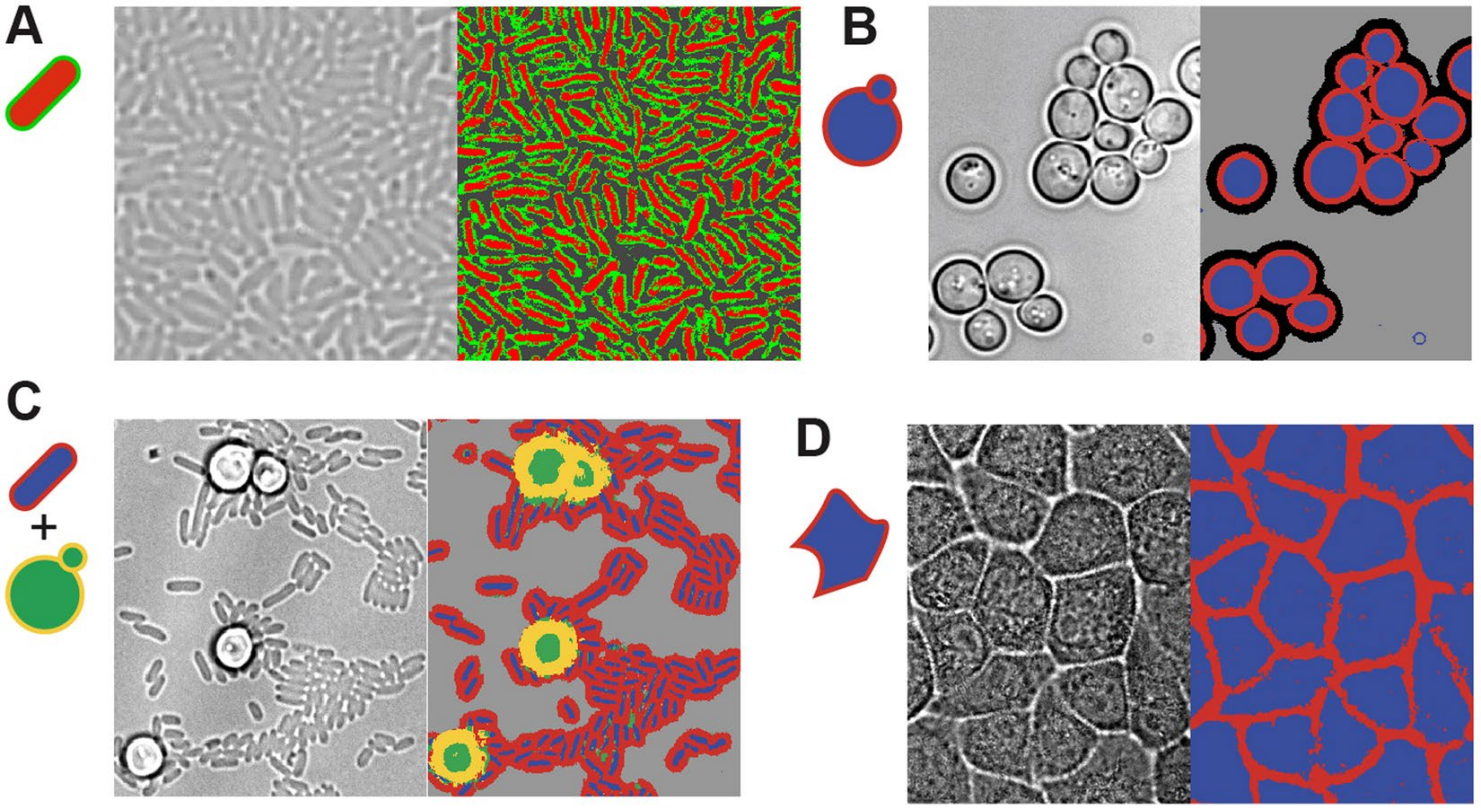
Segmentation of different cell types using the same method. (**A**) *E. coli* cells (~1 µm long, observed with a 100x objective) growing as a monolayer; cell interior and cell contour classes are indicated in red and green, respectively. From left to right: original image; identification results. (**B**) Budding yeast cells (~4 µm wide, observed with a 100x objective) growing in a microfluidic device. Cells are growing as a monolayer and cell interior, cell contour and halo classes are shown in blue, red and black. (**C**) A mixed culture of *E. coli* and budding yeast (observed with a 60x objective). Both cell types can be identified within the same image based only on their focal signatures. (**D**) Monolayer of epithelial HeLa cells (observed with a 60x objective). Cell interior and cell contour classes are shown in blue and red. Adding elementary topological rules (*e.g*. cell contour cannot be inside a cell) and using basic segmentation method (*e.g*. watershed) we obtained good to very good segmentation on yeast, bacteria and mammalian cells (see supplementary text).

Importantly, this method could be applied to different experimental designs. First, we confirmed our method could efficiently identify single bacteria in a dense monolayer of *E. coli* (instead of a few lines of cells) grown between a glass slide and an agar pad (Fig. 3A). We then showed that the method also worked well to identify yeast cells, even though budding yeast cells are larger than *E. coli* cells (~5 µm vs. ~1 µm) and are round (Fig. 3B, Supplementary Fig. S4). We also successfully segmented a mixture of yeast cells and bacteria (Fig. 3D, Supplementary Fig. S5); the system could distinguish between the two types of cells based on their focal signature. This is a particularly hard task for any standard segmentation algorithm based on morphological features alone, indicating this method could represent an important tool for research on infectious diseases or microbial ecology. Additionally, we succeeded in identifying individual epithelial HeLa cells in a confluent monolayer (Fig. 3C, Supplementary Fig. S6). Mammalian cell segmentation is hard, and although deep learning methods have already demonstrated their potential to segment mammalian cells with complex shapes, it came at the expense of large dataset and long computing time^14^. In our case, a simple machine learning algorithm and limited training datasets were sufficient to enable rapid identification of cells with various shapes in a timescale that allow cell classification to be done on the fly. Importantly, our method showed better classification results (see Supplementary Fig. S7) than the Ilastik classifier^10^, a reference image analysis software, which does not use axial information, but encodes information from neighboring pixels. This suggests that axial information contained in Z-stack contains enough information to be used directly for pixel classification (see Supplementary Text).

After classification, the cellular regions are already identified and the cellular segmentation becomes easier. The use of even simple segmentation methods downstream of the classification step provided good results (see Supplementary Text). We anticipate that advanced segmentation algorithms, such as active contour methods, could further improve the fidelity of cell segmentation and tracking.

## Discussion

The method presented in this manuscript does not require a complex imaging setup, since only a few images (~10) in a *z*-stack are required for robust identification of cells. It comes with a GUI to facilitate drawing of the regions of interest in the training dataset. Importantly, it can identify bacteria, yeast, mixture of yeast and bacteria, and mammalian cells (Fig. 3) with the same workflow. Therefore, it appears as a versatile method that can be used to facilitate complex segmentation problems. Moreover, its performance can be further improved. preliminary analysis using Random Forest and Neural Network (See Supplementary Text, Supplementary Fig. S8) showed comparable classification accuracy than SVM, but at much faster speed. Specifically, Random Forest classification was typically 20 times faster than SVM classification. With a single core processor, a full image classification was obtained in typically a minute with a Random Forest classifier. Neural Network classifier also trains in SVM like times, but the prediction usually takes only a couple of minutes. It becomes possible to perform identification in real-time, at least with respect to the typical timescale of single cell microscopy imaging (*e.g*. several minutes between two frames when observing gene expression by fluorescence microscopy). Of course, the processing time could be also drastically decreased by relying on graphic processor unit (GPU) accelerated libraries. Taken together, we anticipate that this versatile method can be integrated into any image analysis pipeline^15^ and can thus tremendously facilitate cell segmentation and tracking problems.

## Methods

### Cell culture and imaging

Cells were cultured and imaged following standard protocols (*E. coli* were grown in LB at 37 °C, yeast in SC at 30 °C and HeLa cells in MDEM at 37 °C and 5% CO_2_). Unless noted otherwise, *z*-stacks were acquired using an IX71 Olympus equipped with a piezo (PIFOC, PI). This allows precision positioning of the objective at a resolution in the tens of nanometers.

### Z-pixel classification

A graphical user interface (GUI, see supplementary text) was used to simplify training set construction. Images were normalized by performing a standard histogram equalization procedure with 1% loss on the histograms. We used principal component analysis (PCA) to reduce dimensionality of the problem. Only a subset of the main principal component (N < 20) dimensions were used to represent the data used to train the classifiers and later generate predictions. For SVMs, we used a method known as winner-takes-all SVM (WTA-SVM), which has the double advantage of providing a classification score for each class and does not require the experimenter to establish a classification tree. The fit implementation of the SVMs was based on the Matlab *fitcsvm* package, which features automatic hyper-parameter optimization, with a Gaussian radial basis function as a kernel and a hinge loss function. We built a manually labeled set and divided it into two parts by random data subsampling: the first part, consisting of 90% of all data was used to train the SVMs. The remaining 10% was used as an evaluation set. Once a satisfactory SVM set was obtained for a particular classification problem, it was used to process new stacks (captured under similar conditions) for cell identification. Stacks to be analyzed were first scaled to the same dynamic range as the training stacks (the histogram of the entire stack is equalized over the maximum range of the training data type). Then transformed into the principal components base. The best SVM set was then applied to the transformed data, and – for each *z*-pixel – a set of classification scores that correspond to each of the classes the SVMs were trained for was computed. Good to very good segmentations were obtained from the classification maps, as shown in supplementary materials. Classification and segmentation methods are described in details in supplementary materials.

### Data Availability

The Matlab code and datasets generated for this study are available on our GitHub repository (https://lab513.github.io/Zcells/, 10.5281/zenodo.1307765) and on a dedicated Zenodo archive (10.5281/zenodo.1307781).

## Electronic supplementary material

supplementary materials
