# Supplementary material for: Identification of individual cells from z-stacks of bright-field microscopy images
Source: Sci Rep. 2018 Jul 30;8:11455. doi: 10.1038/s41598-018-29647-5 (PMC6065389; doi:10.1038/s41598-018-29647-5)
Supplement: Supplementary file 1 — supplementary materials [file 41598_2018_29647_MOESM1_ESM.pdf]

Supplementary Information:

Identification of individual cells from *z*-stacks of bright-field microscopy images.

Jean-Baptiste Lugagne[1,2,*], Srajan Jain[1], Pierre Ivanovitch[1], Zacchary Ben Meriem[1,3], Clément Vulin[4], Chiara Fracassi[1,2], Gregory Batt[2,5] and Pascal Hersen[1,*]

1 Laboratoire Matière et Systèmes Complexes, UMR 7057 CNRS & Université Paris Diderot, 10 rue Alice Domon et Léonie Duquet, 75013 Paris, France.

2 Inria Saclay – Ile-de-France and Université Paris Saclay, 1 rue Honoré d'Estienne d'Orves, Bâtiment Alan Turing, Campus de l'Ecole Polytechnique, 91120, Palaiseau, France

3 Laboratoire Biologie et Dynamique des Chromosomes, CNRS UMR 7212, Hôpital Saint-Louis, Paris, France.

4 ETH, Swiss Federal Institute of Technology, Zurich, Switzerland.

5 Institut Pasteur and CNRS, C3BI - USR 3756, 25-28 Rue du Docteur Roux, 75015, Paris, France.

*Correspondence and requests for materials should be addressed to Jean Baptiste Lugagne (jean-baptiste.lugagne@inria.fr) and Pascal Hersen (*pascal.hersen@univ-paris-diderot.fr*).

1. Supplementary Material and Methods

Here, we provide technical details related to implementation of the classification method. The code is accessible from a GitHub repository (https://github.com/Lab513/Zcells).

Z-pixels

In this step, we aim to classify focal signatures (see Figure 1) from *z*-stack images acquired by imaging the focal planes around the cells' imaging plane. The focal signatures (*z*-pixels) are the pixel illumination levels acquired at each focal plane. In practice, *z*-stacks were acquired using a IX71 Olympus chassis equipped with a piezo-driven motorized objective nosepiece along the vertical axis. This allows precision positioning at a resolution in the tens of nanometers. The method was also successfully tested without piezo-driven positioning, using a lower camera resolution (512 × 512), and at different magnifications (60x, 100x objectives).

Defining classes in the reference image

A graphical user interface (GUI, see Figure ST1) was designed in Matlab to simplify training set construction. This interface allows the experimenter to build a training set based on any number of *z*-stacks, and enables the creation of any number of different classes. When constructing new training sets, the experimenter must take care when defining classes. Indeed, although a hinge loss function is applied to make the training more resistant to erroneous outliers in the dataset, the experimenter must take extra care to not mislabel data when creating the training set. Although this might seem obvious, it is easy for an experimenter to start labeling data a certain way in one part of the set, but end up labeling it in another way by the time they reach the end of the tedious process of creating training datasets. As a typical case, when labeling cell contours and the cytoplasm in bacteria, the limit between the two classes is hard to visualize and, to a large extent,

arbitrary. It is therefore preferable to leave a wide margin between the two classes when identifying *z*-pixels to ensure the two classes will be well-separated in the feature space. At the very least, when establishing a new set, the experimenter/expert should periodically check how they labeled at the beginning of the process to avoid drifting too much. A major component of the classification performance depends on the quality of the training dataset, and while initial trials might be performed on roughly labeled datasets and still produce decent results, performance is improved by careful training set construction.

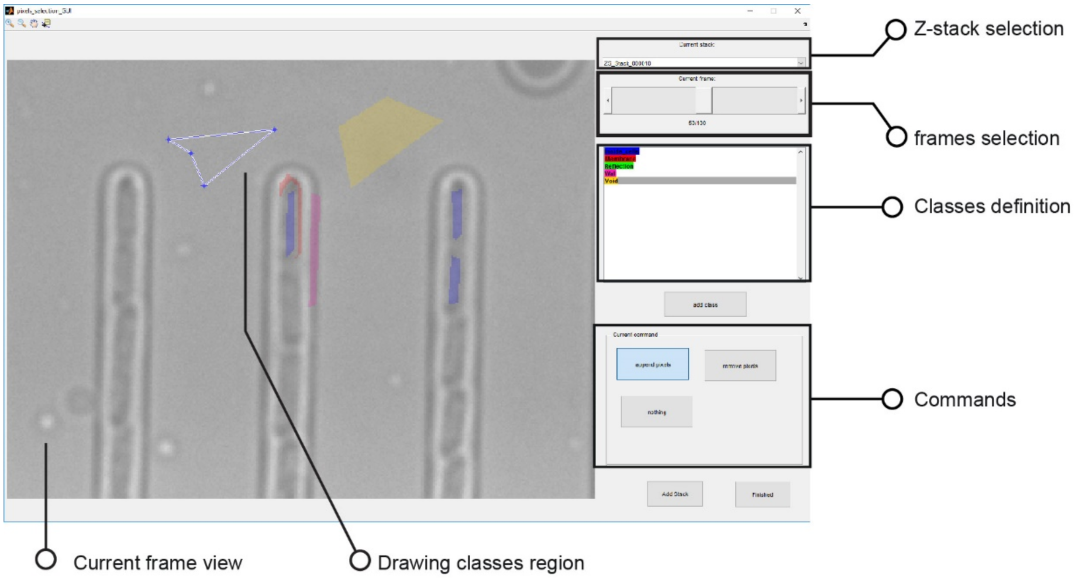

Figure ST1. Matlab graphical user interface (GUI) for training set construction. Several *z*-stacks can be loaded and any number of classes can be created and edited. The user can add and remove regions in the images, which will then be extracted from the stacks and concatenated into a training set and an evaluation set. See Supplementary Movie 1 for a video guide of how to use the GUI.

Data normalization and focus invariance

Images are normalized by performing a standard histogram equalization procedure with 1% loss on the histograms. This is carried out for all of the training stacks and, once the SVMs are trained, on all new *z*-stacks as well. Additionally, a *z*-shift in the focal signatures along the *z*-axis can appear because of auto-focusing errors. As the training *z*-stacks are acquired around the focal plane

at which the cells are in focus, the frames are taken always at the same distance from the central in-focus frame. That means that the features of the training signatures are acquired at specific points around the in-focus point. However, auto-focusing is not always perfect, or the specimen can become tilted. This can lead to a shift of the signature in the *z*-axis. In this case, the shape of all signatures in the stack will appear drastically different to the SVMs. To overcome this problem, we trained the SVMs on shifted data: *z*-stacks for training were acquired at high resolution, and then subsampled for the frames we needed in the stack to train for in-focus data (Figure ST2). Then, an artificial *z*-shift was applied by re-subsampling for frames with a shift in *z*. Subsamples were acquired for an arbitrary shift range and the SVMs were trained on all these data, i.e., the in-focus data and out-of-focus data. This procedure not only made the *z*-pixel classification more robust to auto-focusing errors – up to the point where the autofocus could be so wrong that cells could not be identified anymore in the reference frame and yet *z*-pixels could still be identified properly – but it also made the results more accurate in the absence of auto-focusing errors. We think the shifting procedure works as a sort of data-augmentation method that makes the SVMs better at generalizing new observations. Another advantage of this method is that, with the exception of the shifting range that is decided at the beginning and can be seen as the range of robustness to autofocusing error, the algorithm does not require any arbitrary parameters to be decided upon by the user and no knowledge of signal or image processing is required to correct focusing errors.

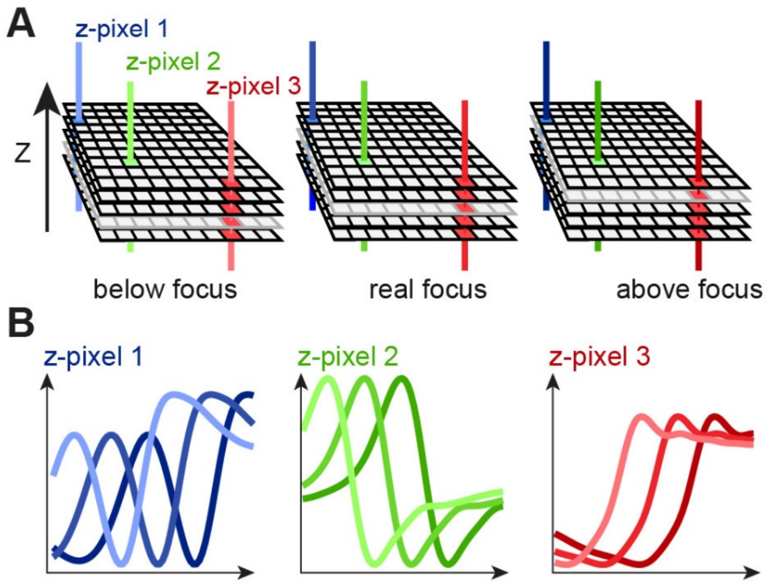

Figure ST2. Correcting a shift of the signature in *z*. A. The signatures extracted from the *z*-stack are artificially shifted by changing the reference of the in-focus frame (in grey). This procedure mimics what happens when erroneous auto-focusing shifts the acquired stack. **B.** SVMs are trained on shifted variants of the same signatures, which increases the robustness of classification.

Each frame in the stacks can be pre-processed with an arbitrary number of image operations. The functionality is implemented such that any image processing operation, of any complexity, that can be written as a Matlab function can be used. A number of common image processing operations are pre-implemented in our code, but the user can pass any custom function they have written to the algorithm and it will be applied to all frames in the stack. The pre-implemented preprocessing functions are:

- **Intensity,** which simply extracts pixel intensity values (i.e. no operation is performed on the frame),
- **Gradient**, which can apply either the Sobel, Prewitt, Roberts, Central or Intermediate image gradient operators to the image,
- **Local average**,
- **Disk average**,
- **Gaussian average**,

- **Laplacian**
- **Laplacian of Gaussian operation**.

Those operations are performed using the functions implementing them in the Matlab Image Processing Toolbox. By default, the software only uses the “Intensity” information, and unless otherwise specified all the results presented in this paper were obtained with only this information. In addition to these, any native or custom image processing function that uses an image as an input and produces an image of identical size as an output can be specified by the user to be applied to each frame in the stack prior to training and prediction. Those preprocessing operations can be used to improve classification accuracy and reduce training or classification time. Because the space of possible preprocessing operations that can be applied is virtually infinite, we could not conduct a systematic study of the impact of the operations on classification accuracy or computation time. However heuristic selection of preprocessing operations shows some reduction of prediction time for some of our classification problems. The time required to compute the preprocessing operations on the stack prior to SVM prediction may in some cases cancel the gains made on prediction time.

Feature extraction and principal component analysis

Dimensionality was reduced using a feature extraction algorithm. We used principal component analysis (PCA), which was performed by computing the set of orthogonal unit eigenvectors of the covariance matrix of the data and transforming the input data into this new space. In other terms, the set of orthogonal axes along which the input data varies the most are computed as linear combinations of the original axes, which are called principal components, and the data is represented in this new base. We performed PCA on the training dataset to obtain the principal component coefficients, which allowed us to transform the data into the principal components

space. In order to reduce dimensionality, only a subset of the first *N'* principal component dimensions are used to represent the data used to train the SVMs (or other classifiers) and later generate predictions. As a rule of thumb, we set *N'* so that the amount of variance accounted for by the first *N'* components was at least 95% of original variance, but not if this meant *N'* was greater than 20 to ensure we would not train the classifiers on extremely high-dimensionality data.

SVM classifier.

As SVMs are binary classifiers, the most common way of handling multiclass data identification is to fit as many SVMs as classes, and then run all SVMs on each signature at the prediction step and take the best result. While other more computationally efficient techniques based on hierarchical classification trees exist, we used a method known as winner-takes-all SVM (WTA-SVM), which has the double advantage of providing a classification score for each class and also does not require the experimenter to establish a classification tree, which can be difficult. The fit implementation of the SVMs was based on the Matlab *fitcsvm* package, which features automatic hyper-parameter optimization, with a Gaussian radial basis function as a kernel and a hinge loss function. To reduce the training time, the fitting of the SVMs for each class was parallelized. Fitting was performed until all SVMs converged (typically 15 minutes to 1 hour).

Evaluation of identification of classes

We built a manually labeled set and divided it into two parts by random data subsampling: the first part, consisting of 90% of all data was used to train the SVMs. The remaining 10% was used as an evaluation set. The evaluation set was used to compare the results of the SVMs for different parameters (number of frames in the stacks, shift-correction range, number of principal components) by running the classification of the SVMs on the evaluation set and then comparing

it to the labels established by the user. The best SVM was selected and used for classification of newly acquired *z*-stacks.

Class prediction and confidence map

Once a satisfactory SVM set has been obtained, it is used to process new stacks (captured under similar conditions) for cell identification. When a new stack is acquired, it is first scaled to the same dynamic range as the training stacks (the histogram of the entire stack is equalized over the maximum range of the training data type). The stack is then transformed into the principal components base by multiplying the *z*-pixels by the principal coefficients from the previously described feature extraction step. The best SVM set is then applied to the transformed data, and – for each *z*-pixel – a set of classification scores that correspond to each of the classes the SVM was trained for is produced. As all *z*-pixels are independent from each other and their classification does not depend on the classification of other pixels, parallelization of the prediction process is trivial. Processing time can be further optimized by defining regions of interest in the image and not classifying regions of the image that do not contain any relevant information.

The SVM raw scores are used to establish a confidence map of image classification. We applied the *softmax* function (see below) to the SVM classification data for each result, and picked the score of the best class as the classification "confidence". The *softmax* function is commonly defined as:

$$s_i(C^p) = \frac{e^{c_i^p}}{\sum_k e^{c_k^p}},$$

with C^p being the vector of the SVM classification scores $(c_1^p, \ldots, c_m^p)$ of the *m* SVMs for each of the *m* classes in the training set, for a *z*-pixel *p* of coordinates (*x*,*y*). $s_i(C^p)$ is the softmax function

evaluated for class *i* on the classification scores of *z*-pixel *p*. We call $S^p = \left(s_1(C^p), \dots, s_m(C^p)\right)$the vector containing the *m* softmax evaluations of each class for *z*-pixel *p*. The *softmax* function restricts the scores to the interval [0, 1], where the initial SVM classification scores could be any element of R. The second interesting property of the *softmax* vector is that the sum of its elements $\sum_k s_k\,(C^p)$ equals one. For these reasons, the S^p vector can be interpreted as a probability distribution, and $s_i(C^p)$ as the probability that pixel *p* is of class *i*. The S^p vector can also be seen as a sort of composition of the signature of *z*-pixel *p* in terms of the archetypal signatures for each of the classes. The confidence map M is constructed from the *softmax* vectors of the corresponding *z*-pixel *p* as the highest *softmax* score for that *z*-pixel:

$M(x, y) = \|S^p\|_\infty$. The confidence maps inform the experimenter or a downstream segmentation algorithm of the reliability of the different parts of the classified image. In particular, the class map M_i defined as $M_i(x, y) = s_i(C^p)$ of a specific class *i* can be used to improve segmentation.

Random Forests classification

Another type of classifier that can be used is Random Forests (RFs) classifiers. RFs hold a theoretical advantage over SVMs in that they can do multi-class prediction and therefore do not require a score-comparison mechanism like “Winner-Takes-All” selection, thus reducing computation time. In practice, while our algorithm was developed for SVMs-based classification, random forest can significantly reduce classification time when used instead of SVMs. The version of the random forest classification algorithm used in this study is the same as the canonical Random Forest method described in [1]. Although classification quality does not seem to be dramatically improved by the use of random forests over support vector machines (SVM), the reduction in prediction time is substantial and therefore should be considered as a better classifier for our method. We allow for the same hierarchical class construction as described for SVMs, with parent

and children classes. Overall, except for classifier-specific parameters, the two versions of our proposed software function in exactly the same ways in appearance, with the same GUIs and scripts. The training sets are also interchangeable.

Neural Network Classification

We also tried to use a neural network to classify z-pixels. We used a 5-layer feed-forward neural network with 4 layers of tunable weights. The first and second hidden layers each have 26 neurons, and the third and fourth hidden layers have 32 neurons each. The input to the neural network are exactly the same z-pixel signatures as what we used for the SVMs classifiers. The network architecture was implemented using Tensorflow and Python. For the training, firstly, PCA is performed on the z-pixel signatures (which may or may not have been appended with the extra features), and some dimension reduction is done, keeping the first 25 components. Then the network is trained using the Adam Optimizer for around 1000 iterations, with a learning rate of 0.1. The training usually takes a few minutes without any explicit parallelization being used. Prediction on a stack takes a few seconds up to a minute or two at worst, for the 1392x1040 images that we used. All the settings mentioned are quite flexible, and can be further fine-tuned to enhance performance.

2. From pixel identification to cellular segmentation

The method described here enables robust classification of cells, and if we only keep relevant classes, we obtain a segmented image showing where cells are located. This first segmentation result can (and should) be improved to ensure that the cell contours are closed and that adjacent cells are correctly separated. To this end, typical cell segmentation techniques can be used downstream of the classification output. Importantly, this cell segmentation becomes much easier and robust, since the different parts of the image are already identified. Also, the classification

scores and confidence map can be used to improve segmentation. Here, we only describe how to proceed with elementary image processing methods. In particular, we did not need to apply complex methods such as active contours. Hence, no assumptions on the shape of the cells were made. Using morphology based methods (active contours) may improve the segmentation performance if chosen properly, but these techniques are beyond the scope of this article.

Basic segmentation

A two-step water shedding technique was applied to the classified image. Firstly, the different water shedding objects/regions are seeded as the "cell interior" regions and then spread into the "cell contour" regions until they connect, thus delimiting the borders of each cell. This procedure produced perfect segmentation of bacterial cells aligned in a microfluidic device and excellent (> 90%) classification on monolayers of bacteria and yeast cells, even when both of these cell types were present in the same culture (see Figure 3).

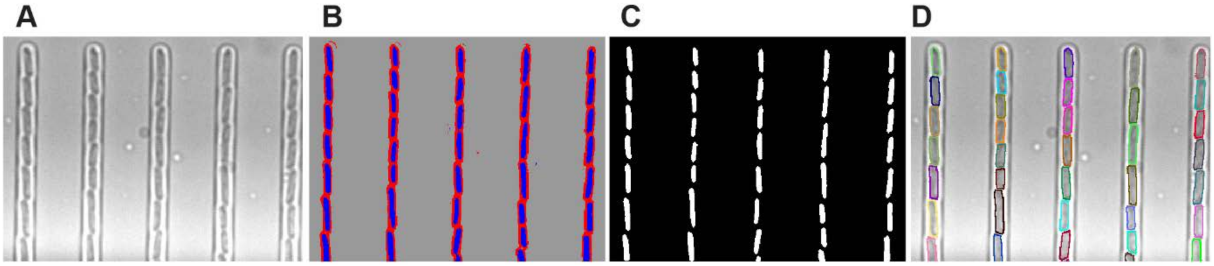

Figure ST3. Use of a seed-based water shedding method to segment bacteria from the classification map. Classes other than cell interior and cell contour are removed and re-labeled as empty. Only regions of the class "interior cell" that are within a "contour" are used as seeds, and a morphological opening with a radius of two pixels is applied to clean the image. The seed-based water shed algorithm is then applied to expand the seeds and segment single cells.

Using class definitions and topological rules

When segmenting a mixture of yeast and bacteria, we added the topological assumption that the class "yeast interior" should be almost entirely surrounded by the class "yeast contour". We filtered

out regions where these classes did not respect this rule, then used the previously described water shedding procedure to segment yeast cells. Then, we considered the rest of the image. The scores of the yeast classes were removed from the C^p classification score vectors to form the new $C^{p\prime}$. The softmax vectors $S^{p\prime}$ were then re-computed for those new classification scores. The *z*-pixels that were classified as part of the two removed classes, but which did not end up being part of the final identified yeast cell regions are re-labeled as the next most-probable class in $S^{p\prime}$. This way, *z*-pixels that were wrongly labeled as part of a yeast cell are re-labeled as other classes, in most cases for the better. Water shedding is then applied again to segment the bacteria. The advantages of this method are that it can be used to segment images in which a mixture of cells is present and to discriminate between different cell types, a result that has so far eluded the field of single cell segmentation.

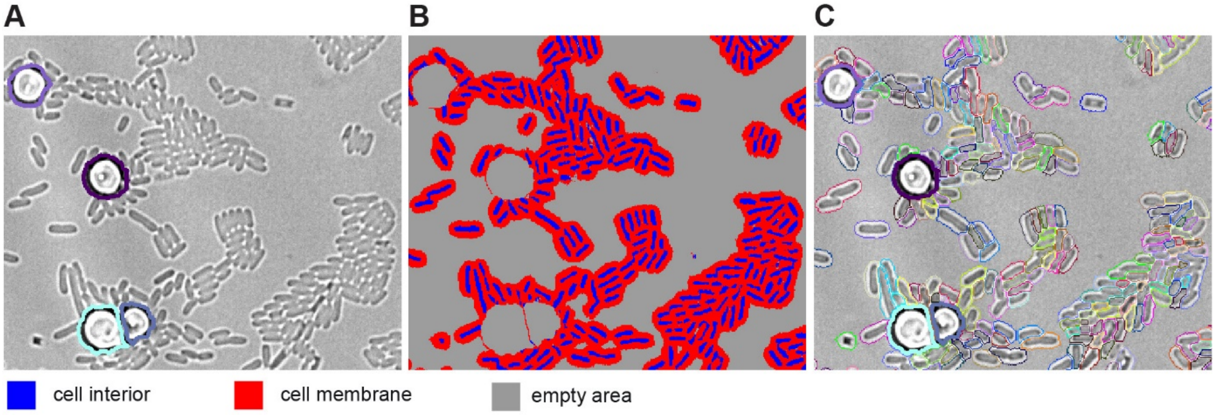

Figure ST4. Using topological rules to segment a mixture of yeast and bacteria. A mixture of yeast and bacteria was processed (see Figure 3 and Supplementary Figure 5). To ensure yeast were properly classified, a topological rule was used to filter misclassified pixels (yeast interior must be encircled by yeast contour). The yeast cells were then segmented via a simple watershed algorithm. Once yeast cells were identified, the yeast classes were removed and the mislabeled elements are re-classified as the next highest-scoring class. Water shed algorithm was then applied again to segment bacteria.

Using a confidence map to improve segmentation

In the case of identifying cells with a more complex shape, such as epithelial cells, the "cell contour" regions in the classified images do not completely encircle all of the cells in the image. In this case, instead of using the *z*-pixels that were strictly identified as the "contour", we can use the class confidence map for the "contour" to identify which *z*-pixels are most likely to be the contour of the cells. The first step in the process is to obtain a rough estimate of the cell centers by eroding the cell objects using a structuring element of variable size. This step is crucial, because the size of the structuring element is the only parameter that is set arbitrarily by the experimenter. Too large a structuring element will remove small cells, but if the structuring element is too small it may not separate cells that are connected in the classified image. From these reduced cell regions, the seeds are expanded back into the confidence map until they connect. As an example, excellent segmentation was achieved for the stack presented in Figure ST5-A, with only minor errors such as removal of the cells on the outer parts of the image (upper right). In other cases, such as the image shown in Figure ST5-E, the segmentation was not perfect and the image was partially under-segmented, though this result is still superior to a typical single image-based algorithm.

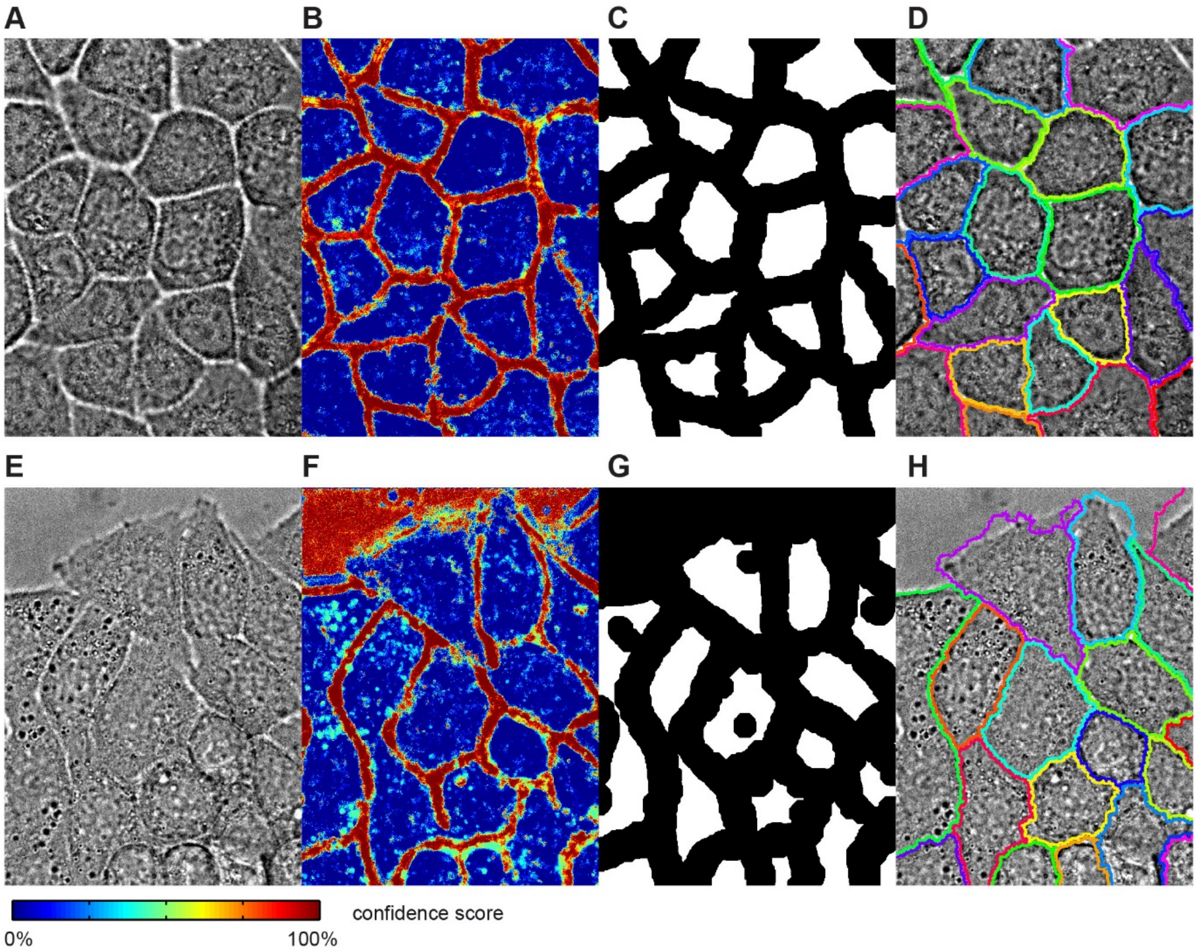

Figure ST5. Using the confidence scores to improve segmentation. Here, we use the confidence map, and not the classification map, to segment a monolayer of epithelial HeLa cells (**A** and **E**). In both examples, the classification does not completely close the contour of the cells. Seeds are identified from the lowest-scoring elements of the "contour" class confidence map (**B** and **F**) and morphologically eroded to partition cells. The seeds are then expanded back into the "contour" confidence map until they all connect (**C** and **G**). This method uses information from the classification confidence instead of morphological rules. However, perfect segmentation is not always obtained; as shown in **E**-**H**, the leftmost cell region is probably two different cells, but is not identified as such. Indeed, seed identification relies on arbitrary parameters, and the experimenter's selection of these parameters can lead to under- or over-segmentation.

References

[1] Breiman, L. Random Forests. Machine Learning 45, pp. 5–32, 2001.

Figure S1

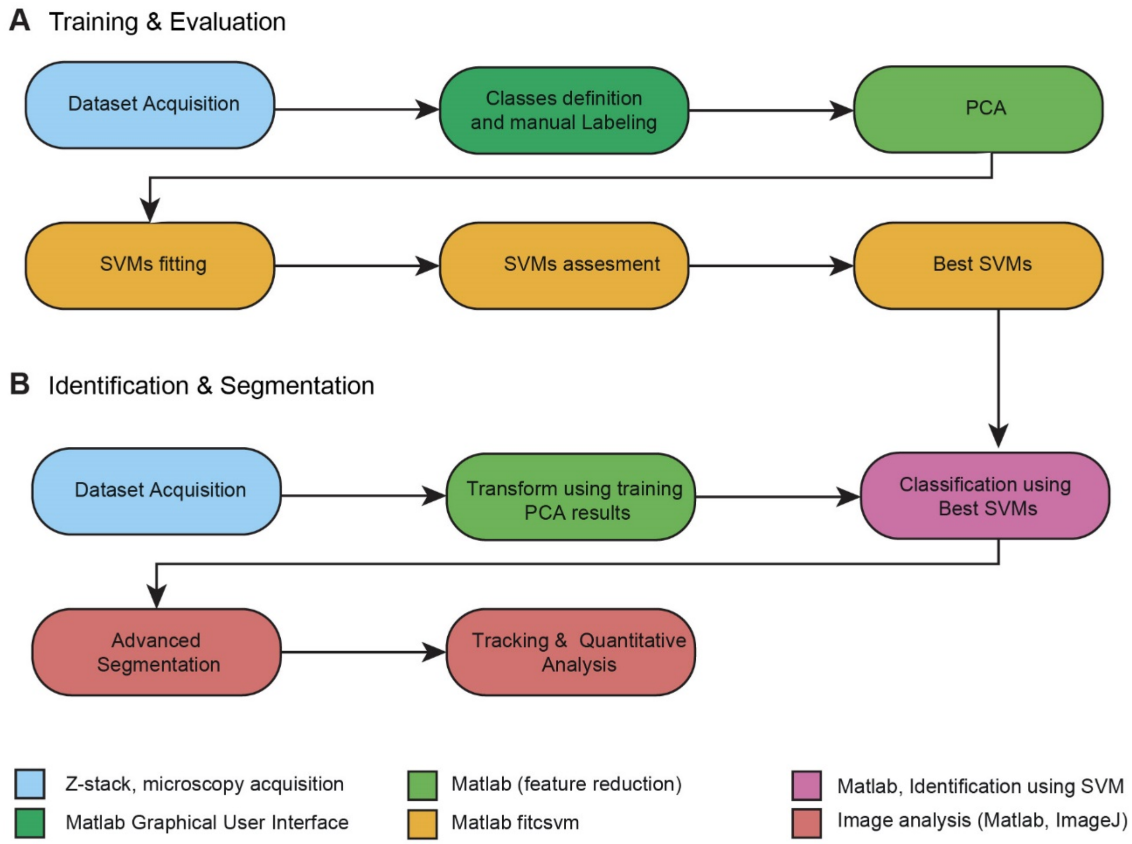

Supplementary Figure 1. Processing images requires training of the classification algorithm (A) and then use of the algorithm to classify new datasets (B). **A. Training a dataset** requires (1) acquisition of one or several *z*-stacks of the cells of interest, (2) opening the training dataset in the graphical user interface, defining relevant classes and identifying corresponding regions of interest (See Figure 1 for an example of how classes are drawn on a training dataset). Once the dataset is correctly labeled, the training algorithm is used to (3) reduce the dimensionality of the problem using principal component analysis (PCA), thus drastically reducing the number of dimensions while preserving maximal original information in the data. On this reduced dataset, a number of SVMs are fitted, each with different sets of parameters (kernel function, misclassification, dimensions tradeoff, maximum number of support vectors…). (4) The classification performance of each SVM is then assessed on the evaluation set, and the best SVM can then be used for identification, or the experimenter can go back to step 3 and improve the performance of the SVM. To improve time of prediction, other classifiers should be used, such as Random Forest and Neural Network. **B. Identification and segmentation of cells** is performed on new, unlabeled data. Data are transformed into the new feature space using the results of the previous PCA. Then, the focal signature of each pixel is classified using the best SVM identified in the training step. Usually, classes include the cell interior and cell contour, which trivializes cell segmentation. Advanced cell segmentation, cell tracking and quantitative analysis can be performed based on the results of pixel identification.

Figure S2

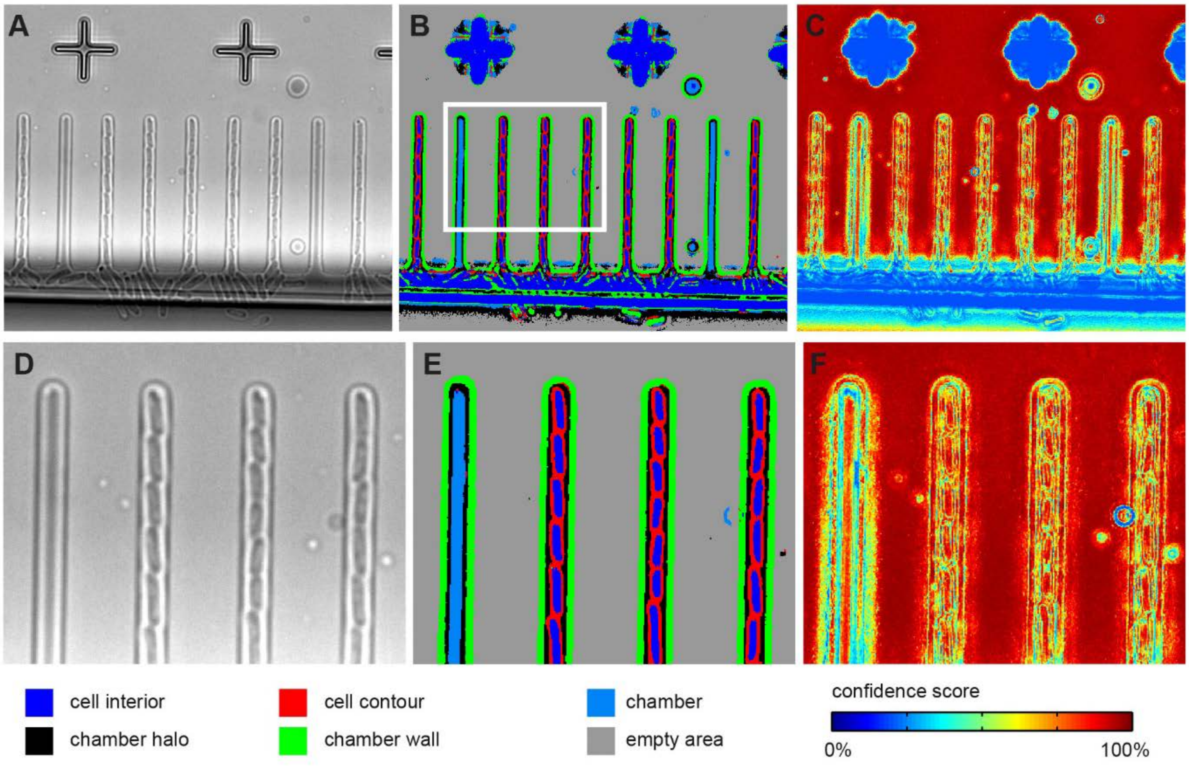

Supplementary Figure 2. Identification results for *E. coli* cells aligned in a special microfluidic device. A stack of 100 frames with 0.1µm *z*-steps was acquired using a 100x oil objective. The SVM was trained to identify six classes. (1) The cell interior (dark blue). (2) The cell contour (red), defined as the outer part of the cell. While the distinction between the cell interior and "cell contour" is not strictly necessary and a single class for the entire cell gives correct identification of cells, this partition of the cell simplifies subsequent segmentation (see Supplementary Text). (3) Empty microfluidic chambers (light blue); (4) chamber halo (black), separating the cells from the (5) microfluidic chamber wall (green) and (6) empty regions (grey). **A.** In-focus image of the bacteria. **B.** Results of the identification. **C.** Confidence score. **D-F.** High-power magnification images of the same images. Since the training set was built with a restricted region of interest in the vicinity of the chambers, the classification is not relevant for the top or bottom edges of the picture. Otherwise, the different regions in the image are correctly labeled, except for a few errors caused by imaging artifacts. On the confidence map, the *softmax* score (see Supplementary Text) of the classification is represented as a heat map. We can see confidence drops in the areas of the image the algorithm was not trained for. The confidence score also drops at the boundary between different regions of the image. This information can be used to improve segmentation (see Supplementary Text).

Figure S3

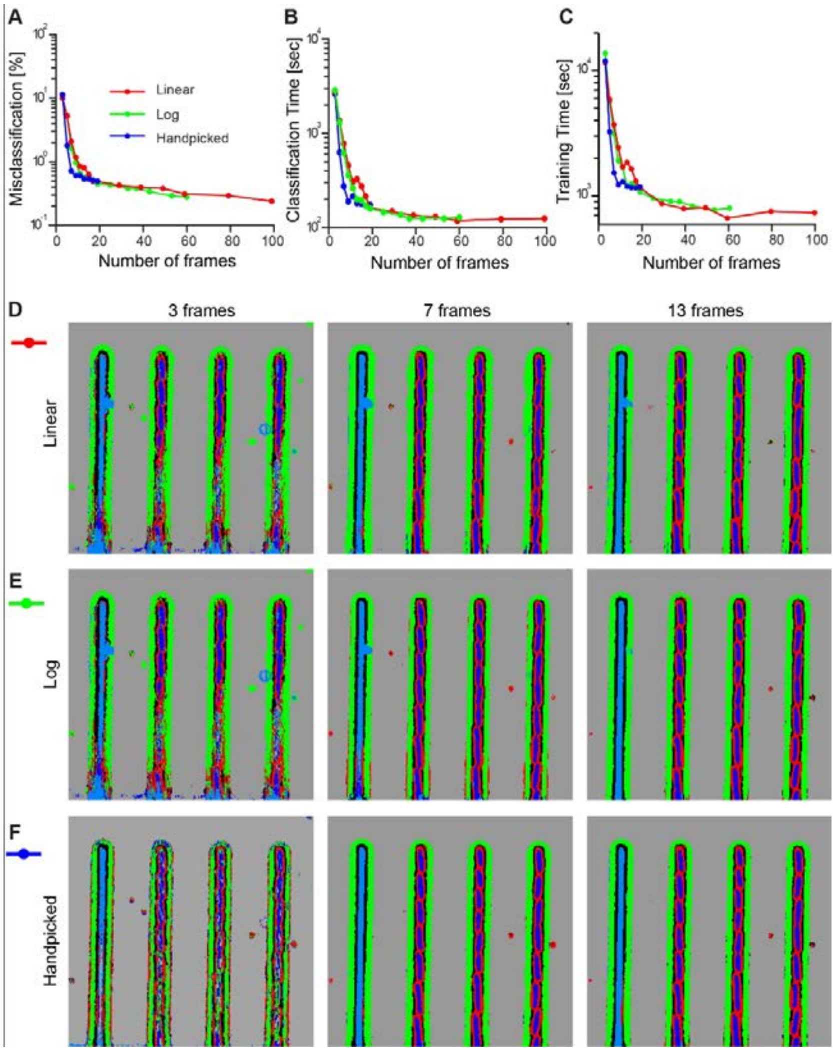

Supplementary Figure 3. The classification method has good performance, even with a small number of frames per stack. We used a training dataset of *E. coli* cells growing in lines in the microfluidic device to examine the precision of the training procedure for different sub-samplings of the original 100-frame stacks. We also varied the method of sub-sampling, testing linear subsampling, meaning frames are sampled with a constant *z*-step between them; logarithmic subsampling, in which the *z*-step increased logarithmically the further away the sampling plane is from the in-focus image; and the hand-picked method, a manual method where the user choses the frames in the stack that seem to be the most informative. **A.** Number of classification errors as a function of the number of frames used for training the algorithm. **B.** Time required to classify a full stack. **C.** Time required to train a full stack. Training was performed without parallelization (**C**), while classification (**B**) was parallelized on CPU over 20 cores. Performance increases dramatically as the number of frames increases from 3 to 20. Above 20 frames, the gains in performance are marginal. **D**-**F**. Classification results for the (**D)** linear, (**E)** logarithmic and (**F)** hand-picked methods over different numbers of frames (from left to right: 3, 7, 13 frames). At 7 frames per stack, the results of the hand-picked method are already good enough for high quality segmentation (see Supplementary Text).

Figure S4

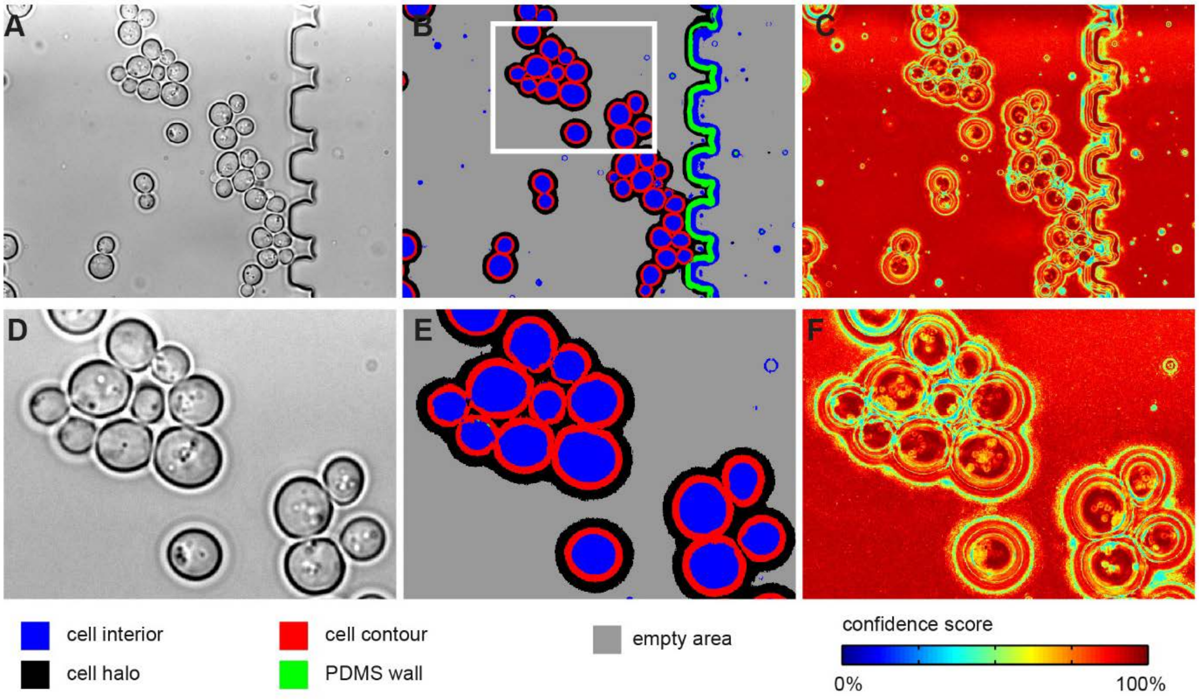

Supplementary Figure 4. Identification results for a monolayer of *S. cerevisiae* cells in a microfluidic device. A stack of 100 frames with 0.1 µm *z*-steps was acquired using a 100x oil objective. The SVM was trained to identify five classes: (1) the cell interior (dark blue); (2) the cell contour (red), defined as the black ring surrounding cells on bright field images; (3) the cell "halo", the white rings around cells; (4) the microfluidic chamber walls (green), seen on the right of the image; and (5) empty regions (grey). **A.** In-focus image of the yeast cells. **B.** Results of identification. **C.** Confidence score. **D-F** High-power magnifications of the same images. Classification of the yeast cells images produced very few errors, which considerably simplifies the problem of yeast cell segmentation. Note that the PDMS wall class is sometimes identified as the cell interior. This misclassification can easily be corrected during the segmentation step by adding topological rules (*e.g.* cell interior must be encircled by cell contour).

Figure S5

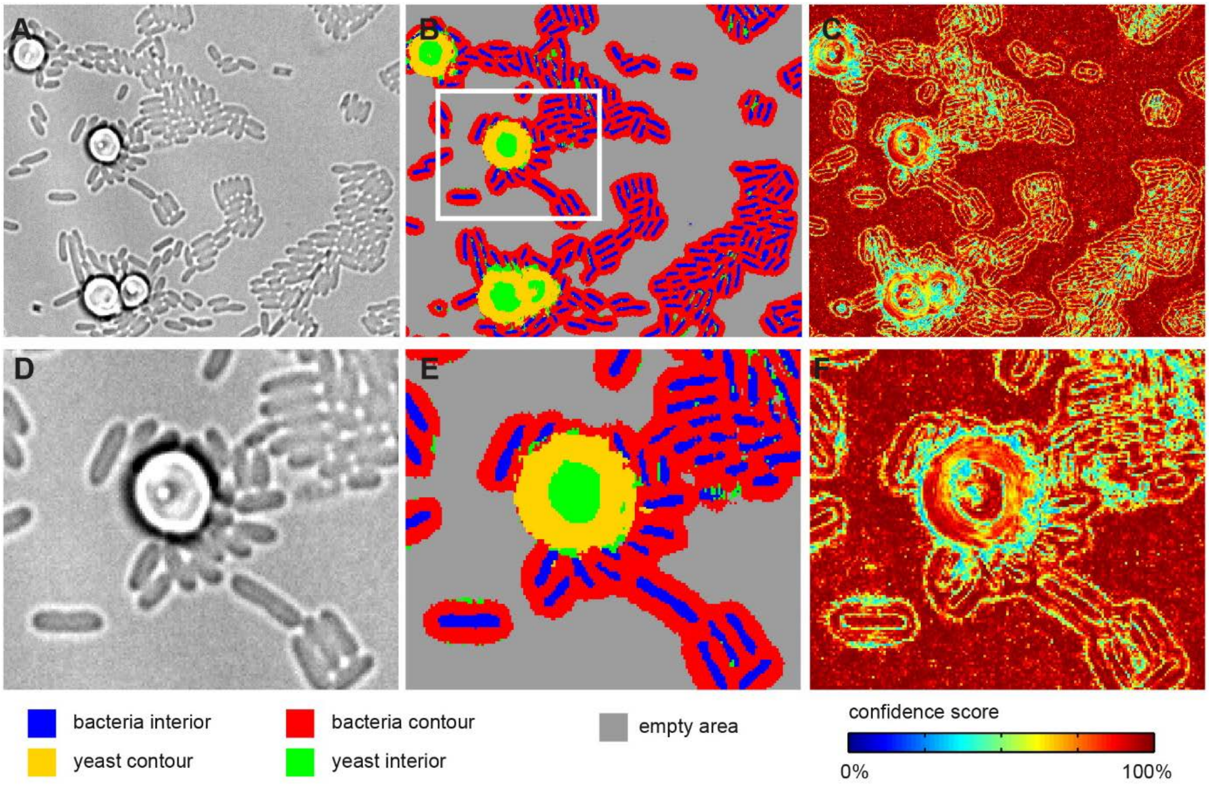

Supplementary Figure 5. Identification results for a mixture of yeast (*S. cerevisiae*) and bacteria (*E. coli*). Stacks of 35 frames with 0.3 µm *z*-steps were acquired in bright-field mode using a 60x oil objective. The SVM was trained to identify five classes: (1) the bacterial cell interior (dark blue); (2) the bacterial cell contour (red); (3) the yeast cell contour (yellow); (4) the yeast cell interior (green); (5) empty regions (grey). **A.** In-focus image of yeast cells. **B.** Results of the identification. **C.** Confidence score. **D-F.** High-power magnifications of the same images. The training set was trained on the same stack the classification was run on. The stack image was split into four sub-regions of equal size, three of which were used for training and the fourth for classification. The stack also contained a smaller number of frames (35), which reduced classification quality (see Figure 2 and supplementary figure 3). As expected, the confidence score drops at the interface between yeast cells and bacteria, but the quality of the classification is still sufficient to proceed to segmentation (see Supplementary Text). Importantly, it is possible to distinguish between the two types of cells based on bright-field images alone.

Figure S6

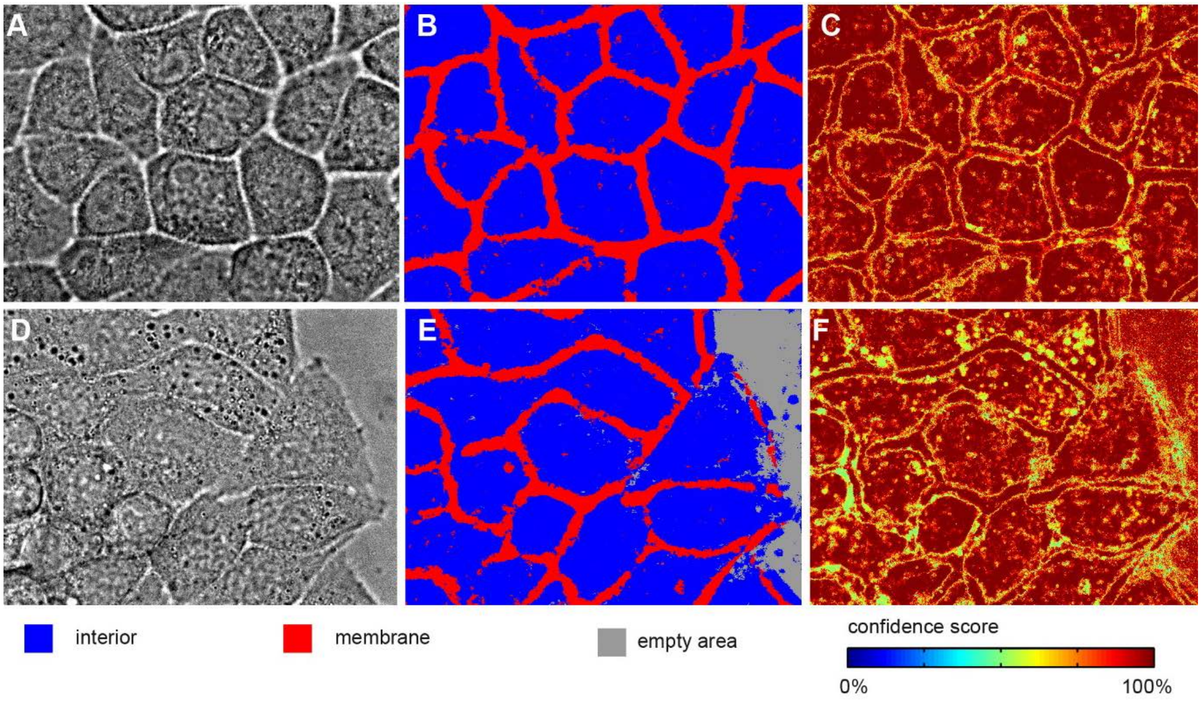

Supplementary Figure 6. Identification results for a monolayer of epithelial HeLa cells. Stacks of 150 frames with 0.3um *z*-steps were acquired with a 60x oil objective in transmitted light. The SVM was trained to identify three classes: (1) the cell interior (dark blue); (2) the cell contour (red, membrane); and (3) empty regions (grey). **A.** In-focus image. **B.** Results of the identification. **C.** Confidence score. **D-F**. Same as **A-C,** for a different *z*-stack. The classification result is of excellent quality and the cells are almost segmented already. In **D,** cell boundaries are not as well-delineated as in **A**, and classification confidence is lower at the interface between the empty region and cells. This may be due to the fact that cells at the periphery are more spread-out than the cells in the center of the monolayer. Adding classes to account for cells at the periphery may improve the segmentation process and help to identify the various morphologies of the cells (i.e. spread or compacted).

Figure S7

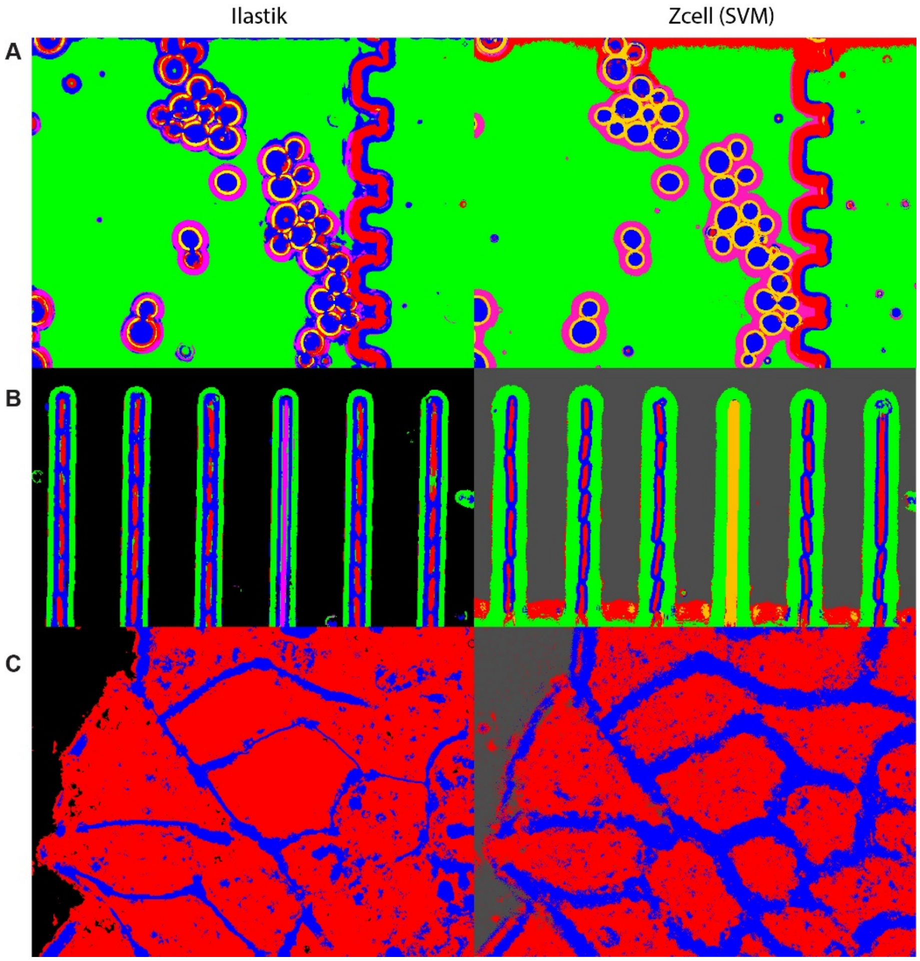

Supplementary **Figure 7**. **Comparison of our method with Ilastik classification results.** Colors show similar classes. Ilastik classifications are shown on the left, our method results on the right**. A.** *Yeast* cell classification. Our approach gives better results for adjacent cells and there are less confusion with other classes.**B.** *E. coli* cells grown in a "mother machine" microfluidic device. Our method is better at identifying pixels at the contour periphery. **C.** Mamalian cells. Ilastik does not perform very well compared to the zcell method.

Figure S8

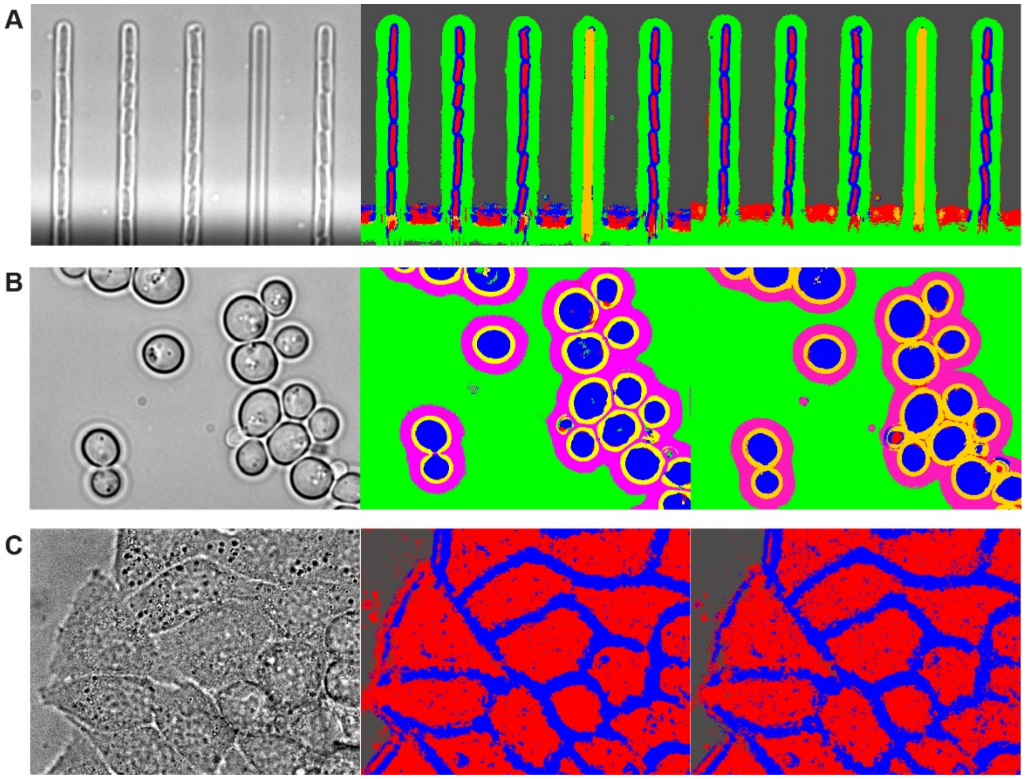

Supplementary Figure 8. Comparison between SVMs, Random Forests and Neural Network classifications. **A.** Close-up of prediction results for *Escherichia coli* cells in a “mother machine” microfluidic device. From left to right: in focus image, Random Forest classification, SVM classification. **B.** Close-up of prediction results for yeast cells**.** From left to right: in focus image, neural network classification, SVM classification. **C.** Prediction results for mammalian cells. From left to right: in focus image, Random Forest classification, SVM classification.
